# Positron Emission Tomography in Animal Models of Tauopathies

**DOI:** 10.3389/fnagi.2021.761913

**Published:** 2022-01-10

**Authors:** Lei Cao, Yanyan Kong, Bin Ji, Yutong Ren, Yihui Guan, Ruiqing Ni

**Affiliations:** ^1^Institute for Regenerative Medicine, University of Zurich, Zurich, Switzerland; ^2^Changes Technology Corporation Ltd., Shanghai, China; ^3^PET Center, Huashan Hospital, Fudan University, Shanghai, China; ^4^Department of Radiopharmacy and Molecular Imaging, School of Pharmacy, Fudan University, Shanghai, China; ^5^Guangdong Robotics Association, Guangzhou, China; ^6^Institute for Biomedical Engineering, ETH Zurich and University of Zurich, Zurich, Switzerland

**Keywords:** Alzheimer’s disease, tau, animal model, positron emission tomography, FTD (fronto-temporal dementia), neurotransmitter, neuroinflammation

## Abstract

The microtubule-associated protein tau (MAPT) plays an important role in Alzheimer’s disease and primary tauopathy diseases. The abnormal accumulation of tau contributes to the development of neurotoxicity, inflammation, neurodegeneration, and cognitive deficits in tauopathy diseases. Tau synergically interacts with amyloid-beta in Alzheimer’s disease leading to detrimental consequence. Thus, tau has been an important target for therapeutics development for Alzheimer’s disease and primary tauopathy diseases. Tauopathy animal models recapitulating the tauopathy such as transgenic, knock-in mouse and rat models have been developed and greatly facilitated the understanding of disease mechanisms. The advance in PET and imaging tracers have enabled non-invasive detection of the accumulation and spread of tau, the associated microglia activation, metabolic, and neurotransmitter receptor alterations in disease animal models. *In vivo* microPET studies on mouse or rat models of tauopathy have provided significant insights into the phenotypes and time course of pathophysiology of these models and allowed the monitoring of treatment targeting at tau. In this study, we discuss the utilities of PET and recently developed tracers for evaluating the pathophysiology in tauopathy animal models. We point out the outstanding challenges and propose future outlook in visualizing tau-related pathophysiological changes in brain of tauopathy disease animal models.

## Introduction

The microtubule-associated protein tau (MAPT) locates intracellularly and is composed of six isoforms, classified into 4-repeat (4R) and 3-repeat (3R) species (Lee et al., 2001; Spillantini and Goedert, 2013). Tauopathy diseases include Alzheimer’s disease (AD), primary tauopathy such as progressive supranuclear palsy (PSP), corticobasal degeneration (CBD), and frontotemporal dementia (FTD) with Parkinsonism linked to chromosome 17 and Pick’s disease. In AD, amyloid-beta (Aβ) and tau interact synergically in the brain and trigger a complex cascade of biochemical and cellular processes, resulting in neurodegeneration (Busche and Hyman, 2020; Chang et al., 2021b). Tau has been an important target in therapeutics development for AD and primary tauopathies. Immunotherapies semorinemab, gosuranemab (Boxer et al., 2019**;** Ayalon et al., 2021**;** Grossman, 2021**;** Mullard, 2021**;** Novak et al., 2021), antisense oligonucleotides (DeVos et al., 2017), and aggregation inhibitors are at different stages of clinical trials (Congdon and Sigurdsson, 2018). Transgenic animal models recapitulating human tauopathy have enabled understanding of disease mechanisms and facilitated the development of treatment strategies (Albert et al., 2019**;** Roberts et al., 2020**;** Ayalon et al., 2021). Varieties of transgenic mouse lines with mutations*on MAPT* gene including P301S (PS19), P301L (JNPL3, rTg4510, pR5) (Lewis et al., 2000**;** Ramsden et al., 2005**;** Santacruz et al., 2005**;** Yoshiyama et al., 2007**;** de Calignon et al., 2012), knock-out hTau (Andorfer et al., 2003), and knock-in (Hashimoto et al., 2019; Saito et al., 2019) mouse models as well as transgenic rat models (Filipcik et al., 2012) have been developed. In addition, 3 × Tg mice and TgF344-AD rats harbor both the Aβ and tauopathy have been widely used (Oddo et al., 2003**;** Cohen et al., 2013). Recent advances in molecular imaging using PET and MRI have provided valuable insights into the time course of disease pathophysiology in tau animal models, including tau, neuroinflammation, and structural and functional alterations (Ishikawa et al., 2018; Ni et al., 2018; Tagai et al., 2020), thus providing a blueprint for tauopathy disease clinical study.

## Tau Imaging

**FIGURE 1:**
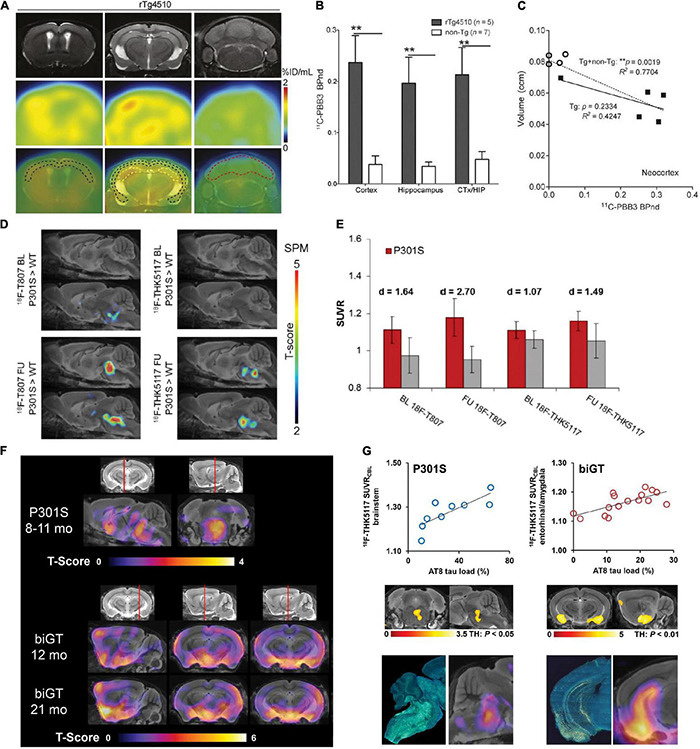
*In vivo* microPET tau imaging in mice **(A)** Representative T_2_-weighted MR, PET using [^11^C]PBB3 and PET/MR images of coronal brain sections of 9-month-old rTg4510 mice showing neocortical, hippocampal, and cerebellar VOIs (black, yellow, and red outlines, respectively). PET images were generated from averaged dynamic data at 30–60 min after injection of [^11^C]PBB3. **(B)** [^11^C]PBB3 binding potential in each VOI calculated by simplified reference tissue model with cerebellum as reference tissue and brain volume measured using structural MRI data including calculation of non-displaceable binding potential for neocortex and hippocampus (CTX/HIP). **p < 0.01, rTg4510 vs. non-transgenic mice. **(C)** Correlation between [^11^C]PBB3 non-displaceable binding potential and volume of neocortex and hippocampus in transgenic (^∙^) and non-transgenic (°) mice. **p < 0.01, for correlations in the transgenic plus non-transgenic group (Tg + non-Tg, dotted lines) and the transgenic group only (Tg, solid lines). Reproduced from Ni et al. (2018) with permission Society of Nuclear Medicine and Molecular Imaging. **(D)** Statistical parametric mapping (SPM) are depicted upon a MRI mouse atlas and extracerebral voxels are masked. The *t*-score threshold of 2 complies a significance threshold of 0.01 uncorrected. **(E)** Bar graphs show group mean relative standard uptake value (SUVR) of P301S (red) and WT (gray) mice for baseline and follow-up PET measurements of [^18^F]T807 and [^18^F]THK5117. Error bars indicate SD and effect sizes are given by Cohen’s *d*. Sagittal slices (median and 0.6 mm paramedian) show voxel-wise SPM between transgenic P301S and WT mice at baseline (BL) and follow-up (FU) for [^18^F]T807 and [^18^F]THK5117. Reproduced from Brendel et al. (2018) with permission from Frontiers SA. **(F)** Mean voxel-wise z score maps in sagittal and coronal planes of [^18^F]THK5117 binding for groups of aged P301S vs. pooled WT mice and biGT mice vs. pooled WT mice. Results of 2-sample *t*-test are expressed as z score maps projected on MRI mouse atlas (gray scale). **(G)** Validation of [^18^F]THK5117 small-animal PET results by immunohistochemical AT8 staining *in vitro* for P301S and biGT mice. Top row shows correlation plots of tau load (%) in corresponding AT8-stained areas with [^18^F]THK5117 SUVR. Middle row depicts linear regression between tau load (%) and small-animal PET SUVR images projected on MRI mouse atlas. Bottom row illustrates AT8-stained sections from single mice along with their individual SPM-derived z score maps (projected on MRI mouse atlas). Reproduced from Brendel et al. (2016) with permission Society of Nuclear Medicine and Molecular Imaging.

Different types of tau inclusions in AD and primary tauopathies have been observed. Neuropil thread, neurofibrillary tangles are observed in AD; Oligodendroglial coiled bodies and argyrophilic threads are common in PSP and CBD. For the glial tau inclusions, tufted astrocytes in PSP, and astrocytic plaques in CBD are observed (Lee et al., 2001). The cerebral tau load assessed by PET using various tau imaging tracers associates with brain regional atrophy assessed by using structural MRI and cognitive impairment in patients with AD (La Joie et al., 2020; Ossenkoppele et al., 2020, 2021; Vogel et al., 2021), CBD, and PSP (Robinson et al., 2020; Tagai et al., 2020; Whitwell et al., 2020). Tau spreading and misfolding follow a disease-specific brain region-dependent pattern first in the entorhinal cortex, hippocampus in human (Wegmann et al., 2019), and in transgenic mouse brain (Clavaguera et al., 2009). The onset and severity of the pathology and brain regions of atrophy vary among the different strains. The rTg4510 line develops tauopathy at a young age (4–5 months) and showed atrophy in both the cortex and hippocampus. In contrast, hTau mice mainly show atrophy in the cortex and PS19 mice demonstrate pathology mainly in the brain stem and spinal cord (Lewis et al., 2000; Andorfer et al., 2003; Ramsden et al., 2005; Santacruz et al., 2005; Yoshiyama et al., 2007; de Calignon et al., 2012). MicroPET imaging of tau in tauopathy rodent models has contributed to the development of novel PET tracers, understanding of disease mechanism, and monitoring of treatment effect. Several tau tracers have been tested in tau mouse models including 2,6-disubstituted naphthalene derivative [^18^F]FDDNP (Teng et al., 2011), pyridinyl-butadienyl-benzothiazole 3 derivatives [^18^F]PM-PBB3 (APN-1607), [^11^C]PBB3, [^11^C]mPBB5 (Maruyama et al., 2013; Ishikawa et al., 2018; Ni et al., 2018; Barron et al., 2020; Tagai et al., 2020), arylquinoline derivatives [^18^F]THK523 (Fodero-Tavoletti et al., 2011), [^18^F]THK5351 (Moreno-Gonzalez et al., 2021), [^18^F]THK5317 (Filip et al., 2021), [^18^F]THK5117 (Brendel et al., 2016; Chaney et al., 2021), pyridoindole derivative [^18^F]flortaucipir (Brendel et al., 2018), and lansoprazole derivative [^18^F]NML (Shao et al., 2012; Fawaz et al., 2014; Table 1). Using [^18^F]THK523, Brendel et al. (2018) showed significantly higher tracer retentions in brains of 6-month-old rTg4510 mice compared with non-transgenic mice or PS1/APP mice with Aβ pathology, indicating specific detection of tau. Brendel et al. (2016) and Chaney et al. (2021) demonstrated that PET using (S)-[^18^F]THK5117 showed higher tracer uptakes in PS19 biGT mice [glycogen synthase kinase-3β (GSK-3β) × P301L tau] and TgF344 rats compared to non-transgenic littermates, respectively (Figures 1F,G). However, Eckenweber et al. (2020) reported that in PS19 mice at 6 month-of-age, (S)-[^18^F]THK5117 was not able to detect the tau accumulation, although presence of tau accumulation was validated by using *ex vivo* immunohistochemical staining. Moreno-Gonzalez et al. (2021) recently showed an elevated regional [^18^F]THK5351 PET signaling in brain of PS19 tau mice, correlating with histological levels of tau. However, both the binding assays as well as *in silico* experiment showed that [^18^F]THK5351 had the limitation of off-target binding to monoamine oxidase B (Ng et al., 2017; Murugan et al., 2019). The most widely used first-generation tau tracer [^18^F]flortaucipir was reported to showed greater difference compared to [^18^F]THK5117 in tracer retention in APPswe × P301L vs. non-transgenic mice (Brendel et al., 2018; Figures 1D,E). However, *ex vivo* binding and autoradiography from two other studies showed lack of detection using [^18^F] flortaucipir in rTg4510 mice (Marquié et al., 2015; Ni et al., 2018). PET using [^11^C]PBB3 imaging for tau has been demonstrated in PS19 (Chang et al., 2021b) and rTg4510 mice (Ishikawa et al., 2018; Ni et al., 2018; Takuwa et al., 2020; Figures 1A–C). PS19 mice showed a mainly brainstem and spinal cord tracer retention, while in rTg4510 mice the retention was observed in the cortex and hippocampus in line with the *ex vivo* validation (Maruyama et al., 2013). An age-dependent increase in [^11^C]PBB3 signal was observed in 7–11-month-old rTg4510 mice, consistent with neuropathological observations (Ni et al., 2018). In addition, the tau load inversely correlated with neocortical volumes assessed by T2 structural MRI, indicating an association between tau and neurodegeneration (Ishikawa et al., 2018; Ni et al., 2018). The disadvantages of [^11^C]PBB3 include non-negligible binding to Aβ plaques in patients with AD and the short half-life. Thus, the second-generation [^18^F]PM-PBB3 with improved binding properties was developed to overcome the limitations. Similar observations were reported by Tagai et al. (2020) and Weng et al. (2020) using [^18^F]PM-PBB3 in rTg4510 mouse models with increased tracer retention in the cortical and hippocampal regions. Among the other second-generation tau tracers, [^18^F]JNJ-64349311 (Declercq et al., 2017) and [^18^F]PI-6240 (Kroth et al., 2019) have so far been reported in wild-type mice for brain uptake and biodistribution assessment. In addition, several new tau probes are underdevelopment such as [^18^F]IBIPF1 (Kaide et al., 2019), [^18^F]PI-2014 (Gabellieri et al., 2020), [^18^F]PPQ (Lerdsirisuk et al., 2021), [^11^C]LM229 (McMurray et al., 2021), [^18^F]2-phenylquinoxaline derivatives (Zhou K. et al., 2021), antibody-based imaging (Krishnaswamy et al., 2014), and 4R-tau specific tracer [^18^F]CBD-2115 (Lindberg et al., 2021).

**TABLE 1:** Summary of PET imaging in tauopathy animal models.

Target	PET tracer	Animal model	References
Tau	[^11^C]PBB3	rTg4510 mice	Maruyama et al., 2013; Ishikawa et al., 2018; Ni et al., 2018; Takuwa et al., 2020
		PS19 mice	Maruyama et al., 2013; Barron et al., 2020; Fairley et al., 2021; Ji et al., 2021
	[^18^F]APN-1607	rTg4510 mice	Tagai et al., 2020; Weng et al., 2020
	[^18^F]flortaucipir	PS19 mice	Brendel et al., 2018
	[^18^F]FDDNP	3 × Tg rats	Teng et al., 2011
		TgF344 rats	Cohen et al., 2013
	[^18^F]THK-5317	APP/Tau rats	Filip et al., 2021
	[^11^C]THK-5351	P301S mice	Moreno-Gonzalez et al., 2021
	[^18^F]THK-5105	PS19, biGT mice	Brendel et al., 2016, 2018
	[^18^F]THK-5117	TgF334 rats	Chaney et al., 2021
	[^11^C]TH523	rTg4510 mice	Fodero-Tavoletti et al., 2011
	[^11^C]LM229	PS19 mice	McMurray et al., 2021
	[^18^F]NML, [^18^F]LNS	hTau + / + rats	Shao et al., 2012; Fawaz et al., 2014
TSPO	(R)-[^11^C]PK11195	rTg4510 mice	Ishikawa et al., 2018; Chiquita et al., 2019
		3 × Tg mice	
	[^18^F]FEBMP	PS19, rTg4510 mice	Barron et al., 2020; Fairley et al., 2021; Ji et al., 2021
	[^11^C]DAA1106	PS19 mice	Ji et al., 2008
		TgF334 rats	Chaney et al., 2021
	[^18^F]FEDAA1106	PS19 mice	Maeda et al., 2011; Ji et al., 2021
	[^11^C]AC-5216	rTg4510 mice	Maeda et al., 2011; Ishikawa et al., 2018; Takuwa et al., 2020; Ji et al., 2021; Zhou X. et al., 2021
		PS19 mice	Ji et al., 2021
	[^18^F]DPA-714	TgF344 rats	Chaney et al., 2021
	[^11^C]PBR28	5 × FAD, PS19 mice	Mirzaei et al., 2016; Ji et al., 2021
	[^125^I]CLINDE	3 × Tg mice, TgF344 rats	Tournier et al., 2019, 2020
	[^18^F]GE-180	PS19 mice	Eckenweber et al., 2020; Palleis et al., 2021
		TgF344 rats	Chaney et al., 2021
P2Y12R	[^11^C]AZD1283	rTg4510, PS19 mice	Maeda et al., 2021
OATP1C1	[^18^F]2B-SRF101	3 × Tg mice	Kreimerman et al., 2019
OGA inhibitor	[^18^F]MK-8553	rTg4510 mice	Wang et al., 2020
MC-I	[^18^F]BCPP-EF	rTg4510 mice	Barron et al., 2020
MT	[^11^C]MPC-6827	PS19 mice	Sai et al., 2020
α7nAChR	[^18^F]ASEM	TgF334 rats	Chaney et al., 2021
BzR	[^11^C]flumazenil	rTg4510 mice	Shimojo et al., 2020
mGluR5	(E)-[^11^C]ABP688	rTg4510 mice	Shimojo et al., 2020
	[^18^F]FPEB-PET	5 × FAD mice	Lee et al., 2019
CMRglc	[^18^F]FDG	tauVLW mice	de Cristóbal et al., 2014
		5 × FAD mice	Rojas et al., 2013; Macdonald et al., 2014; Franke et al., 2020
		3 × Tg mice	Sancheti et al., 2013; Adlimoghaddam et al., 2019
		hTau mice	Brendel et al., 2019
		Tg601 mice	Hara et al., 2017
		PS19 mice	Eckenweber et al., 2020
Neutrophil	[^68^Ga]PEG-cFLFLFK	3 × Tg mice	Kong et al., 2020
Astrocyte	[^18^F]2B-SRF101, [^11^C]DED	3 × Tg mice	Kreimerman et al., 2019
Nasal neuron	[^11^C]GV1-57	rTg4510 mice	Van de Bittner et al., 2017

*α7nAChR, a7 nicotinic acetylcholine receptor; BzR, benzodiazepine receptor; CMRglc, cerebral metabolic rate of glucose; [^11^C]DED, N-(^11^C-methyl)-L-deuterodeprenyl; mGluR5, metabotropic glutamate receptor 5; FDG, fluorodeoxyglucose; MC-I, mitochondria complex-I; MT, microtubule; OATP1C1, organic anion-transporting polypeptide 1C1; OGA, O-linked N-acetylglucosamine (O-GlcNAc)ase; TSPO, translocator protein; SV2A, synaptic vesicle glycoprotein 2A; NML, N-methyl lansoprazole; WT, wild-type.*

## Neuroinflammation Imaging

Neuroinflammation play an important role in patient with AD and other primary tauopathy diseases as well as in animal models of tauopathy (Heneka et al., 2015; Ising et al., 2019; Wu et al., 2019; Linnerbauer et al., 2020; Richetin et al., 2020; Gaikwad et al., 2021; McAlpine et al., 2021; Palleis et al., 2021), featured by disease associated with microglia activation, reactive astrocytes, and activated cytokines such as complement C3 and interleukin-3. Immunohistochemical staining showed that synapse loss and microglial activation precede the appearance of tangles in PS19 mice (Yoshiyama et al., 2007). NLR family pyrin domain containing 3 (NLRP3) inflammasome activation was reported to drive tau pathology (Ising et al., 2019). A marked reduction of homeostatic microglial genes was found by single-cell sequencing of microglia isolated from rTg4510 mice, correlating with the degree of neuronal loss (Sobue et al., 2021). Shi et al. (2021a) and Wang C. et al. (2021) showed that microglia promoted apolipoprotein E-dependent neurodegeneration in tau mice (Shi et al., 2019) and that selective removal of astrocytic apolipoprotein E can protect against tau-mediated neurodegeneration and decrease synaptic phagocytosis by microglia. Microglia activation has been shown to occur preceding the tangle formation in tau mice (Yoshiyama et al., 2007). In addition, microglia was activated to engulf neuron containing tau aggregates, turned hypofunctional after phagocytosis, released the seed component of tau aggregates, and, thus, facilitated the spreading of tau in PS19 mice (Bellucci et al., 2004; Brelstaff et al., 2021). Thus, imaging of neuroinflammation in tauopathy mice offers crucial dynamic pathophysiological information and potential diagnostic parameter (Leng and Edison, 2021).

### Translocator Protein

**FIGURE 2:**
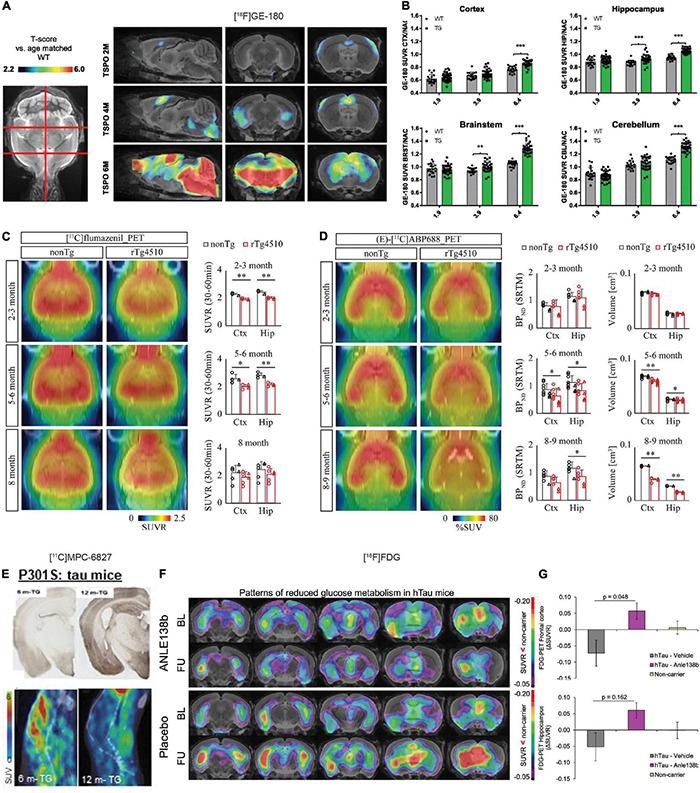
*In vivo* microPET translocator protein (TSPO), synaptic, and metabolic alterations imaging in tauopathy mice. **(A)** Age-dependent exponential increase of 18kDa TSPO expression in different target regions of the brain of P301S tau model mice. n(P301S/WT) = 1.9M, 33/18; 3.9M, 32/17; 6.4M, 29/17. **(B)** Voxel-wise SPM analysis of TSPO expression in the contrast of P301S vs. wild-type mice at different ages. T-score maps are projected upon an MRI template in sagittal and coronal slices. ^***^p < 0.001. Reproduced from Eckenweber et al. (2020) with permission Society of Nuclear Medicine and Molecular Imaging. **(C)** PET assessment of inhibitory synapse with [^11^C]flumazenil in non-Tg and rTg4510 mice at age 2–3, 5–6, and 8 months after peripheral bolus administration of [^11^C]flumazenil. Representative PET images generated by averaging dynamic scan data at 30–60 min are shown. Brainstem was set as reference region. *p < 0.05; ^**^p < 0.01; Student’s *t*-test. **(D)** PET assessment of excitatory synapse with (E)-[^11^C]ABP688 in non-Tg and rTg4510 mice at age 2–3, 5–6, and 8–9 months after peripheral bolus administration of (E)-[^11^C]ABP688. Representative PET images generated by averaging dynamic scan data at 0–90 min are shown. (E)-[^11^C]ABP688 PET was analyzed in cortex (Ctx) and hippocampus (Hip) by simple reference tissue model with cerebellum as reference region. *p < 0.05 (Mann–Whitney *U* test). Reproduced from Shimojo et al. (2020) with permission from Biomed Central Ltd. (Springer Nature). **(E)** Immunohistochemical tau staining in 6-month- and 12-month-old transgenic P301S tau mice with their corresponding dynamic 0–45 min microPET/CT images with [^11^C]MPC-6827 injection. Decrease in [^11^C]MPC-6827 radioactive uptake with increase in tau loads. Reproduced from Sai et al. (2020) with permission from John Wiley and Sons. **(F)** Serial [^18^F]fluorodeoxyglucose (FDG)-PET imaging of relative cerebral metabolism at follow-up (FU) imaging 3 months after late-stage novel aggregation-inhibiting oligomer modulator Anle138b treatment in hTau mice, with reduced baseline compared to control mice. **(G)** Quantification of longitudinal changes in relative FDG uptake (ΔSUVR) indicates normalization of cerebral metabolism in the hTau-treated group, while ongoing decrease in hTau-vehicle group. Baseline (BL) at 14.5 months of age and FU at 17.5 months. Reproduced from Brendel et al. (2019) with permission from Springer Nature AG.

The most widely used neuroinflammation probes are those targeting at 18 kDa Translocator Protein (TSPO), locates on the outer mitochondrial membrane, and overexpressed by microglia during activation (Van Camp et al., 2021; Zhou R. et al., 2021). TSPO PET tracers that have been applied in tau animal models include first generation (R)-[^11^C]PK11195, second generation [^18^F]FEBMP, [^18^F]DPA-714, [^11^C]AC-5216, (^125^I) CLINDE, [^11^C]PBR28, [^18^F]FEDAA1106, and [^11^C]DAA1106 (Ji et al., 2008, 2021; Maeda et al., 2011; Jacobs and Tavitian, 2012; Mirzaei et al., 2016; Ishikawa et al., 2018; Tournier et al., 2019, 2020; Barron et al., 2020; Takuwa et al., 2020; Chaney et al., 2021; Fairley et al., 2021; Leng and Edison, 2021; Zhou X. et al., 2021), and third generation [^18^F]GE-180, etc. (Eckenweber et al., 2020; Chaney et al., 2021; Palleis et al., 2021). However, the first generation [^11^C]PK-11195 has several disadvantages such as high non-specific plasma binding, low signal-to-noise ratio as well as relatively low entrance into the brain and difficulty in quantitative analysis. The second-generation TSPO tracers improved the limitation of [^11^C]PK-11195 and demonstrated favorable binding properties. However, the binding of second-generation TSPO tracers differs greatly in humans depending on the *rs6971* polymorphism in the *TSPO* gene and can be categorized into high-, mixed-, and low-affinity binders (Owen et al., 2012). This polymorphism adds complexity and introduces high variability among subjects. Therefore, the third-generation TSPO tracers are being developed to overcome this limitation. In preclinical TSPO imaging studies, no evidence with respect to *rs6971* polymorphisms has been reported. Microglia activation assessed by using [^11^C]PK11195 PET was reported to co-localize with tau and can predict disease progression and tau accumulation assessed by [^18^F]flortaucipir in patients with PSP (Malpetti et al., 2020, 2021). Takuwa et al. (2020) demonstrated higher [^11^C]PBB3 uptake (tau accumulation) and [^18^F]AC-5216 (microglia activation) in rTg4510 mice at 6 month-of-age compared to wild-type littermates. The regional [^18^F]AC-5216 retention correlated with both the tau level and brain atrophy assessed by T_2_ structural MRI (Takuwa et al., 2020). Eckenweber et al. (2020) reported that [^18^F]GE-180 measures of microglia activation was accompanied by [^18^F]fluorodeoxyglucose (FDG) reduction with increasing age in PS19 mice at 2–6 months. This microglia activation also predicted the increased tau accumulation assessed by immunohistochemistry and deteriorated spatial learning in the Morris water maze (Eckenweber et al., 2020; Figures 2A,B). The specific detection of [^18^F]GE-180 was demonstrated by serial PET during pharmacological depletion of microglia in PS19 mice (Palleis et al., 2021). Barron et al. (2020), Fairley et al. (2021), and Ji et al. (2021) have assessed microglia activation by using [^18^F]FEBMP, which showed higher specificity to microglial TSPO and demonstrated increased microglia activation along with increased uptakes of [^11^C]PBB3 for tau deposits in rTg4510 and PS19 mice.

### Beyond Translocator Protein

The probes utilized so far for microglia activation imaging are not specific for a specific activation status (proinflammatory M1 or anti-inflammatory M2) (Jain et al., 2020). TSPO tracers have several limitations such as diverse cellular sources on both the astrocytes and microglia as well as high non-specific binding level (Nutma et al., 2021). Different targets have been pursued for neuroinflammation imaging toward a clearer activation status and have been evaluated in tauopathy animal models. These include tracers for purinergic P2Y12 receptor [[^11^C]AZD1283], for organic anion-transporting polypeptide 1c1 [[^18^F]2B-SRF101], and for neutrophil infiltration [(^68^Ga) PEG-cFLFLFK] (Kreimerman et al., 2019; Kong et al., 2020; Maeda et al., 2021). P2X7 deficiency has been shown to improve plasticity and cognitive abilities in THY-Tau22 mice (Carvalho et al., 2021). Maeda et al. (2021) showed a distinct response of P2Y12 receptor to tau deposits using [^11^C]AZD1283 by *ex vivo* autoradiography and immunohistochemical staining in rTg4510 and PS19 tau mice. The levels of P2Y12 receptor declined in tau-laden region with increased total level of microglia (Maeda et al., 2021). However, low brain uptake was observed for this tracer, which hinders further *in vivo* usage. Kreimerman et al. (2019) recently reported N-[3-[^18^F]fluoropropyl] sulfonamide [[^18^F]2B-SRF101] as a potential astrocytosis tracer in 3 × Tg mice: a higher [^18^F]2B-SRF101 uptake was observed in the cortex and hippocampus of 3 × Tg compared to control mice, while the N-(^11^C-methyl)-L-deuterodeprenyl (DED) showed a different uptake pattern.

## Metabolism Imaging

### Cerebral Glucose Metabolism

[^18^F]FDG is commonly used in assisting the early and differential diagnosis of AD, FTD, and Parkinson’s disease, of which regional cerebral glucose hypometabolism is present. Most studies report global cerebral glucose hypometabolism in PS19, tauVLW mice as well as in 3 × Tg, 5 × FAD mice with both the amyloid and tau pathologies (Sancheti et al., 2013; Macdonald et al., 2014; DeBay et al., 2017; Hara et al., 2017; Son et al., 2018; Adlimoghaddam et al., 2019; Brendel et al., 2019; Eckenweber et al., 2020; Franke et al., 2020). However, Rojas et al. (2013) reported an increased [^18^F]FDG uptake resulted from glial activation in 5 × FAD mice compared to wild-type mice. Hara et al. (2017) showed cerebral glucose hypometabolism first in the medial septum measured by using [^18^F]FDG PET and spread to the hippocampal dentate gyrus in aged Tg601 tau mice. Brendel et al. (2019) demonstrated a reduced [^18^F]FDG uptake in brain of hTau mice compared to wild-type mice and that treatment using novel aggregation-inhibiting oligomer modulator Anle138b can rescue this reduction in [^18^F]FDG measures at follow-up scan after treatment (Figures 2F,G). Recent study by Xiang et al. (2021) showed that [^18^F]FDG uptake reflected metabolism derived from microglia and that the increased [^18^F]FDG might instead due to microglia activation.

### Mitochondria

Mitochondrial ATP production is crucial in brain bioenergetics and is associated with brain homeostasis, functions, synaptic plasticity, and neurotransmitter processes (Du et al., 2008). The mitochondrial complex 1 (MC-1) plays an important role in the ATP production process, maintains calcium homeostasis, and regulates the apoptosis pathways. Altered MC1 function has been associated with neuronal toxicity, which contributes to the development of various neurodegenerative diseases including AD, FTD, and Parkinson’s disease. Thus, MC-1 has been an attractive target for imaging biomarker indicative of neuronal damage and metabolic changes. As MC-1 locates inside neuron and not microglia or astrocytes, the probe will detect specifically the neuronal metabolism. Several tracers such as [^18^F]BCPP-EF, [^18^F]BCPP-BF, [^18^F]BCPP-EM, and [^18^F]BMS-747158-02 have been developed (Harada et al., 2013; Tsukada et al., 2014). And among these tracers, [^18^F]BCPP-EF shows the sufficient brain uptake and a reversible binding pattern. Terada et al. (2021) recently demonstrated reduced uptake of brain [^18^F]BCPP-EF, associating with [^11^C]PBB3 measures of tauopathy and cognitive performance in patients with mild AD. In addition, the reduction of [^18^F]BCPP-EF in the parahippocampus of early-stage AD may precede cerebral glucose hypometabolism assessed by using [^18^F]FDG (Terada et al., 2020). Barron et al. (2020) and Fairley et al. (2021) showed a reduced [^18^F]BCPP-EF uptake in the forebrain and hippocampus in rTg4510 mice compared to wild-type mice. Moreover, the [^18^F]BCPP-EF signal co-localized with tau accumulation assessed by using [^11^C]PBB3, regional atrophy assayed by structural T_2_ MRI, and negatively associated with microglia activation assessed by using [^18^F]FEBMP in rTg4510 mice (Barron et al., 2020; Fairley et al., 2021).

## Synaptic Neurotransmitter Receptors

Aberrant accumulation of tau, especially the toxic oligomeric type, has been shown to be associated with altered synaptic protein expression, impairment in axonal transport, neurotransmitter deficits (e.g., cholinergic and glutamatergic), and leading to synaptic loss (Lasagna-Reeves et al., 2011; Spires-Jones and Hyman, 2014; Ait-Bouziad et al., 2017; Lo et al., 2019; Chang et al., 2021b). Several receptors have been shown to be involved in the tau-induced neurotoxicity: Tau modulates the N-methyl-D-aspartate (NMDA) receptor-dependent excitotoxicity and depotentiation in mouse models (Miyamoto et al., 2017). M1 and M3 muscarinic receptors have been shown to mediating the tau-induced intracellular calcium increase and alter calcium ion homeostasis (Gómez-Ramos et al., 2008). Oligomeric forms of tau have been shown to impair synaptic function and recognition memory function in mice (Lasagna-Reeves et al., 2011). Neuronal activity, in turn, enhances tau propagation and pathology (Wu et al., 2016). Chang et al. (2021a,b) recently showed that tau reduction reduced the excitation-inhibition ratio and decreased network hypersynchrony. The abnormal accumulation of tau deposits is directly associated with neuronal loss in animal models (Gómez-Isla et al., 1997; Park et al., 2020), gray matter atrophy, and cognitive deficit in AD (Clavaguera et al., 2009; Murray et al., 2011; Nelson et al., 2012; Bejanin et al., 2017; Ossenkoppele et al., 2021). Recent studies have showed a close correlation between postmortem tauopathy and atrophy assessed by structural MRI as well as functional connectivity in patients with CBD and PSP (Spina et al., 2019). Neuronal hyperactivity has been reported to enhance tau spread *in vivo* in tau mice (Wu et al., 2016). Selective disruption of inhibitory synapses led to neuronal hyperexcitability at an early stage of tau pathogenesis in PS19 mice (Shimojo et al., 2020).

### Nicotinic Acetylcholine Receptors

The cholinergic system is important for memory and cognitive function. Impairment in cholinergic signaling, cholinesterase, decreased levels of nicotinic acetylcholine receptors (nAChRs) that was found at an early stage of AD and primary tauopathy diseases (Ni et al., 2013; Murley and Rowe, 2018). Several new α7 nAChR tracers have been developed including [^11^C]NS14492 (Ettrup et al., 2011), [^18^F]DBT-10 (Hillmer et al., 2016), [^18^F]YLF-DW (Wang D. et al., 2021), and [^18^F]ASEM (Horti et al., 2014). In animal models, Chaney et al. (2021) showed using an age-dependent increase in [^18^F]ASEM uptake in the striatum and nucleus basalis of Meynert in the wild-type rats and was higher compared to that in the TgF344 rats at 18 month-of-age. [^18^F]ASEM has also been evaluated in Parkinson’s disease animal model with striatal injection of 6-hydroxydopamine, in which a transient increase in the level of α7 nAChR was detected (Vetel et al., 2020). A comparative PET study comparing [^18^F]ASEM and [^18^F]DBT-10 indicated that [^18^F]ASEM harbored better brain uptake and kinetic behavior in non-human primates (Hillmer et al., 2017). [^18^F]ASEM imaging was reported in patients with mild cognitive impairment (Coughlin et al., 2020), psychosis (Coughlin J. et al., 2018), schizophrenia (Wong et al., 2018), and in healthy aging (Coughlin J. M. et al., 2018). Coughlin et al. (2020) showed an increased cerebral [^18^F]ASEM measure of α7 nAChR in patients with mild cognitive impairment compared to healthy controls.

### Metabotropic Glutamate Receptors

Metabotropic glutamate receptors (mGluRs) play important roles in memory and learning in regulating neuronal cell death and survival (Murley and Rowe, 2018). Among the mGluRs, the mGluR5 subtype has been most implicated in AD and FTD (Lee et al., 2004), with several tracers been developed and evaluated in animal and in human, e.g., [^18^F]FPEB (Mecca et al., 2020), (E)-[^11^C]ABP688 (Treyer et al., 2007), and [^18^F]PSS232 (Sephton et al., 2015; Warnock et al., 2018). Lee et al. (2019) reported a 35% decrease in the cortical and subcortical [^18^F]FPEB binding in 5 × FAD mice at 9 month-of-age compared to 3 month-of-age. Shimojo et al. (2020) demonstrated a reduced level of inhibitory synapse by using [^11^C]flumazenil at 2–3 month-of-age and a reduced level of excitatory synapse by using (E)-[^11^C]ABP688 at 5–6 month-of-age in rTg4510 mice compared to wild-type littermates, preceding tau accumulation and brain regional atrophy (Figures 2C,D). Similarly, Treyer et al. (2020) demonstrated a reduced (E)-[^11^C]ABP688 uptake in the hippocampus and amygdala in patients with AD compared to healthy controls. Mecca et al. (2020) showed an age-dependent reduction of [^18^F]FPEB-PET level in health control cases probably due to tissue loss. However, conflicting result was reported, where a higher level of [^18^F]PSS232 binding was detected in autoradiography using postmortem brain slices from AD compared with healthy controls (Müller Herde et al., 2019).

## Discussion

Recent advances in PET imaging tracers have enabled *in vivo* visualization of the time course of central pathologies in patients with AD and primary tauopathy diseases as well as in disease animal models (Jacobs and Tavitian, 2012). The multiplex molecular, structural, and functional imaging readouts have provided important etiological insights, facilitating the understanding of disease mechanism (Hoenig et al., 2018; Jacobs et al., 2018; Franzmeier et al., 2019; Hanseeuw et al., 2019; La Joie et al., 2020; Vogel et al., 2020). In this study, we summarize several considerations in further PET studies in tauopathy animal models.

1. Improvement in animal models: Recent studies have indicated that genomic disruption in addition to mutant tau led to neuronal loss in the widely used rTg4510 mouse model (Ramsden et al., 2005; Santacruz et al., 2005). Moreover, the C57BL/6 strain background also impacts on the development of tauopathy in the rTg4510 model (Bailey et al., 2014). Tau propagates more quickly when human tau is knocked into the mouse locus (Gamache et al., 2019). New models such as the knock-in mouse model (Hashimoto et al., 2019; Saito et al., 2019) or non-human primate model (Beckman et al., 2021) that better recapitulate the human tauopathy diseases are essential for evaluation of imaging tracers and for understanding the tau-related pathophysiological alterations.
2. Difference in the structure of tauopathy between animal models and human: The mostly widely used P301L and P301S transgenic mouse models, which overexpress human 4R tau, show accumulation of pretangle, hyperphosphorylated tau, and neurofibrillary tangles in the brain parenchymal. However, the tau fibril structure is different in animal models compared to that in human with 4R tauopathy diseases, partly due to difference in seeding potency, posttranslational modification, cell-type specificity, as well as a much shorter disease development period (Narasimhan et al., 2017). Recent cryogenic electron microscopy study further demonstrated the complexity and structural differences in the folding of tau filaments among AD (3R/4R), primary tauopathy such as CBD (4R), PSP (4R), argyrophilic grain disease (4R), and Pick’s disease (3R) (Shi et al., 2021b).
3. Quantification and reference brain region: Different observations across different PET imaging in tauopathy animal models were observed, partly due to the use of different strains and age groups, as the pathophysiology spatial distribution and time course differ among different models. For the quantification of PET tracer uptake, standard uptake value (SUV), percent injected dose per gram (ID%/g), and more advanced reference brain region modeling have been utilized (Kuntner and Stout, 2014). For quantifying tau tracer uptake in animal models, relative SUV (SUVR) was calculated using cerebellum as reference brain region. Difficulty in identifying a suitable reference brain region and high non-specific binding of tracer (such as TSPO tracer) hinder the accuracy of advanced analysis.
4. Emerging synaptic targets: Synaptic loss assessed by PET using synaptic vesicle proteins 2A (SV2A) tracer [^11^C]UCB-J was recently reported in patients with primary tauopathies (Holland et al., 2020) and in AD (Coomans et al., 2021) where a link between tau [assessed by [^18^F]flortaucipir] and SV2A was detected. No study has so far been reported using SV2A PET in tauopathy animal models. In addition, other emerging targets such as o-GlcNAcase (Paul et al., 2019; Wang et al., 2020) and GSK-3β (Liang et al., 2016; Hu et al., 2017; Prabhakaran et al., 2017; Bernard-Gauthier et al., 2019; Varlow et al., 2021) that associated with tauopathy remain to be explored in tauopathy models. Pilot study results from microtubule imaging using novel tracer [^11^C]MPC-6827 in PS19 mice and cynomolgus monkeys showed promising results (Damuka et al., 2020; Sai et al., 2020), where a decrease level of [^11^C]MPC-6827 uptake accompanied by an increased level of tau was detected (Figure 2E).
5. Imaging microglia activation and astrocytosis: There is a lack of imaging tracers for detecting the dynamic phenotypes of microglia, especially the disease-associated microglia with more specific cellular location and activation status (proinflammatory/anti-inflammatory). Several promising targets are currently under investigation such as purinergic P2X7 receptors, P2Y12 receptors (Beaino et al., 2017, 2020; Van Weehaeghe et al., 2020), cyclooxygenase-1 and cyclooxygenase-2 (Ohnishi et al., 2014, 2016; Shukuri et al., 2016), macrophage colony-stimulating factor 1 receptor (Horti et al., 2019; Zhou X. et al., 2021), cannabinoid receptor type 2 (Savonenko et al., 2015; Ni et al., 2019, 2021a), imidazoline-2 binding sites, and receptor for advanced glycation end products (Cary et al., 2016; Luzi et al., 2020). Recent studies highlighted the role of astrocyte in tau pathology and the associated neurodegeneration (Bussian et al., 2018; Ferrer et al., 2018; Richetin et al., 2020; Maté de Gérando et al., 2021; Wang C. et al., 2021; Wang and Ye, 2021). Tau oligomer exposure was shown to trigger the senescence and toxic subpopulation of astrocytes (Gaikwad et al., 2021; Maté de Gérando et al., 2021). For astrocytosis, imaging several tracers targeting at monoamine oxidase-B [e.g., [^11^C]deuterium-L-deprenyl and [^11^C]SMBT-1] (Marutle et al., 2013; Rodriguez-Vieitez et al., 2015, 2016; Schöll et al., 2015; Carter et al., 2019; Bellaver et al., 2021; Ni et al., 2021b) and at imidazoline binding site (I_2_-BS) [e.g., [^11^C]BU99008] have shown promising results (Calsolaro et al., 2021). How the astrocytosis evolves longitudinally in tauopathy animal models remains to be demonstrated.
6. Sex difference: In human, sex modifies APOE ε4 dose effect on brain tau deposition in cognitively impaired individuals. Sex differences in cerebrospinal fluid tau levels and a mediating effect of testosterone were reported (Sundermann et al., 2020). In amyloidosis animal model, higher load of cerebral Aβ level in female compared to male mice and difference in immune system and metabolism have been reported (Eede et al., 2020; Ni, 2021). In tau animal models, the gender influence on the pathophysiology has been less well documented. A recent study showed increased olfactory, motor deficits, and tau pathology in Tau-P301L male mice compared to female mice (Camargo et al., 2021). Imaging of GSK-3β also indicated a sex difference in P301L mouse model with difference in tracer uptake only between male P301L and wild-type mice (Knight et al., 2021). In other studies, no behavioral, structural, and metabolic difference was observed in the P301S (Criver.com, 2021) and P301L mice (Ni et al., 2020; Massalimova et al., 2021). Several histology and behavior studies in 3 × Tg mice have also indicated a higher amyloid load in female mice compared to male mice, while no difference in tau level between groups (Hirata-Fukae et al., 2008; Carroll et al., 2010; Yang et al., 2018). The recent platform such as MODEL-AD provides an excellent platform for comparative studies and further understanding of the phenotype- and sex-related behavioral and pathological differences in the animal models (Oblak et al., 2020; Sukoff Rizzo et al., 2020; Vitek et al., 2020).

In conclusion, PET has provided a systematic non-invasive approaches to probe the spreading of tauopathy and the related neuroinflammatory, metabolic, and synaptic alterations as well as monitoring of treatment effect in tauopathy animal models.

## Author Contributions

LC, YK, and RN wrote the manuscript draft. All authors contributed to the manuscript and approved the final version of the manuscript.

## Conflict of Interest

LC was employed by Changes Technology Corporation Ltd. The remaining authors declare that the research was conducted in the absence of any commercial or financial relationships that could be construed as a potential conflict of interest.

## Publisher’s Note

All claims expressed in this article are solely those of the authors and do not necessarily represent those of their affiliated organizations, or those of the publisher, the editors and the reviewers. Any product that may be evaluated in this article, or claim that may be made by its manufacturer, is not guaranteed or endorsed by the publisher.
